# Evaluation of the laboratory performance of MolecuTech®REBA MTB-MDR kit for the detection of multidrug resistant tuberculosis

**DOI:** 10.1016/j.jctube.2026.100616

**Published:** 2026-05-06

**Authors:** Eun-Soon Son, Ji-im Lee, Ji-Hoi Kim, Eunjin Cho, Mee-Kyung Kee, SoonMog So, Jong Seok Lee

**Affiliations:** a ITRC (International Tuberculosis Research Center), 236 Gaposunhwan-ro, Masanhappo-gu, Changwon-si, Gyeongsangnam-do, the Republic of Korea; b YD Diagnostics (Young Dong Diagnostics), 76 Seo-Ri Ro, Yidong-Myun, Cheoin-Gu, Yongin-Si, Kyunggi-Do, the Republic of Korea

**Keywords:** MDR-TB, MolecuTech®REBA MTB-MDR, Diagnosis, DST

## Abstract

This study evaluated the performance of the MolecuTech® REBA MTB-MDR kit, a molecular diagnostic tool developed by YD Diagnostics, for MDR-TB detection. We assessed 174 clinical *Mycobacterium tuberculosis* isolates. The MolecuTech® REBA MTB-MDR kit performance was compared with the Hain MTBDR*plus* assay and sequencing analysis. Sensitivity, specificity, and concordance for detecting isoniazid (INH) and rifampicin (RIF) resistance were evaluated. The MolecuTech® REBA MTB-MDR kit demonstrated high sensitivity and specificity for both INH (100% and 100%, respectively) and RIF (100% and 100%, respectively) resistance detection. The inclusion of the *ahpC* gene target enhanced INH resistance detection. The automated HybREAD480® system improved workflow efficiency and reduced human error. The MolecuTech® REBA MTB-MDR kit shows promising performance for rapid MDR-TB detection, comparable or superior to the World Health Organization (WHO)-recommended Hain MTBDR*plus* assay. This study showed that the MolecuTech® REBA MTB-MDR kit is expected to contribute to developing effective tuberculosis treatment strategies in clinical settings.

## 1 Introduction

Tuberculosis (TB), a chronic infectious disease caused by *Mycobacterium tuberculosis*, remains a significant global public health concern. According to the World Health Organization (WHO) Global Tuberculosis Report 2024, approximately 10.8 million people fell ill with TB in 2023, with an estimated 1.25 million deaths. This underscores TB's position as one of the leading causes of death from a single infectious agent worldwide, second only to COVID-19 [1], [2]. Multidrug-resistant tuberculosis (MDR-TB) is defined as TB that is resistant to at least INH and RIF, the two most potent first-line anti-TB drugs [2]. MDR-TB can develop through acquired resistance due to inadequate drug regimens or improper adherence to treatment, or through primary infection with MDR-TB strains. While drug-susceptible TB typically requires 6–9 months of treatment, MDR-TB necessitates a prolonged treatment duration of 18–20 months [3]. Therefore, rapid and accurate diagnosis is crucial for early intervention and effective treatment. The global burden of MDR-TB remains a significant challenge. In 2024, an estimated 390,000 people developed MDR/RR-TB (rifampicin-resistant TB), with only about 40% of these cases being diagnosed and reported [1], [2]. This gap in detection highlights the urgent need for improved diagnostic capabilities [4]. Traditionally, MDR-TB has been diagnosed using culture-based drug susceptibility testing (DST), which can take 3–4 months to yield results. This delay in diagnosis can lead to inappropriate treatment and further transmission of drug-resistant strains. Therefore, the development of rapid and accurate diagnostic methods for MDR-TB remains a critical priority in global TB control efforts [5]. Molecular diagnosis of MDR-TB involves detecting genetic mutations associated with drug resistance. For rifampicin (RIF) resistance, over 95% of cases are associated with mutations in the 81-base pair Rifampicin-Resistance Determining Region (RRDR) of the *rpoB* gene. isoniazid (INH) resistance is linked to mutations in three different genetic regions: about 66% occur in the *katG* codon 315 region, 21% in the *inhA* promoter, and 5% in the *oxyR-ahpC* intergenic region [6], [7]. YD Diagnostics has developed the MolecuTech® REBA MTB-MDR (REBA) V2, an improved version of their line probe assay (LPA) for detecting INH and RIF resistance. This assay utilizes reverse blot hybridization assay (REBA) technology, which is recommended by WHO for TB diagnosis. The REBA MTB-MDR kit simultaneously detects resistance to RIF and INH by targeting mutations in the *rpoB*, *katG*, *inhA*, and *ahpC* intergenic regions. The test results are automatically interpreted using the MolecuTech HybREAD480® automatic device system, enhancing efficiency and reducing human error. This study aimed to evaluate the sensitivity, specificity, and concordance of the MolecuTech®REBA MTB-MDR (REBA) V2 assay in detecting drug resistance, comparing it with the widely used Hain MTBDR*plus* assay and sequencing analysis. Using 174 strains from the International Tuberculosis Research Center, selected based on their Mycobacterial Growth Indicator Tube (MGIT) phenotypic drug susceptibility testing (pDST) results, this study aimed to assess the performance of this new diagnostic tool. For a more comprehensive evaluation, results will be verified using the Hain test and sequencing.

## 2 Methods

### 2.1 Mycobacterial isolates

*M. tuberculosis* isolates cultured from sputum samples of patients with active pulmonary TB were obtained from the National Masan Hospital (NMH), Republic of Korea (ClinicalTrials.gov identification number NCT00341601). This observational cohort study was reviewed and approved by the NMH Ethics Review Board. A total of 174 *M. tuberculosis* isolates comprising MDR-TB and fully susceptible strains based on previous pDST results and sequencing data were selected for inclusion in the study. Clinical *M. tuberculosis* isolates were sub-cultured on Lowenstein-Jensen (LJ) media and incubated at 37℃ for 4–6 weeks [8].

### 2.2 Phenotypic drug susceptibility testing

pDST using the MGIT960 system (BD Diagnostics, Franklin Lakes, NJ, USA) was performed according to the manufacturer’s instructions. Drug concentrations used to determine resistance were 0.1 μg/mL for INH, 1.0 μg/mL for RIF, 1.0 μg/mL. To each 7 mL MGIT tube, 0.8 mL of MGIT 960 Growth Supplement and 0.1 mL of the drug stock solutions were aseptically added, and finally 0.5 mL of the test inoculum was added. For each isolate, a growth control (GC) tube with Growth Supplement and without drug was included. For GC, the inoculum was prepared by pipetting 0.1 mL of the test inoculum with 10 mL of sterile water to make a 1:100 dilution; 0.5 mL of GC inoculum was added to a drug-free MGIT tube. All of the inoculated tubes were placed into the BACTEC MGIT 960 instrument on the same day of inoculation. The relative growth ratio between the drug containing tube and drug-free GC tube was determined by the system’s software algorithm. If the relative growth in the drug-containing tube was equal to or exceeded that of the GC tube, the isolate was considered drug resistant; if the relative growth was less than in the GC tube, the isolate was considered drug susceptible. The instrument did the final interpretation and 84 reported the susceptibility results automatically [9], [10].

### 2.3 Genomic DNA preparation

Mycobacterial genomic DNA was extracted from mycobacterial colonies growing on LJ medium by suspending one loop of mycobacterial colony in 200 μL distilled water and incubating at 100℃ for 10 min. The supernatant containing DNA was collected and stored at −20 ℃ until use.

### 2.4 MolecuTech® REBA MTB-MDR kit

The MolecuTech® REBA MTB-MDR kit is a new LPA for the detection of MDR-TB using the MolecuTech HybREAD480®instrument, an automated LPA system comprising the entire LPA process up to the interpretation of the results. Using the REBA MTB-MDR kit, polymerase chain reaction (PCR) amplification of different genes for drug resistance can be performed using multiplex PCR under the same conditions. The REBA MTB-MDR strip comprises 19 probes (13 wild-type and 6 mutant-type-specific probes) for the detection of RIF, INH (e.g., RIF and INH resistance) (Supplementary Fig. S1). Before the evaluation of this kit, an analytical experiment was performed by testing a mixed experimental sample (50% of the sample was mutated with respect to wild-type). The limit of detection (LoD) was found to be 100 copies/reaction using serial dilution of the quantified extracted H37Rv. Clinical genomic DNA (5 μL) was used for amplification, which was performed using GeneAmp 9700 (Applied Biosystems, Waltham, MA, USA). The multiplex PCR conditions included an initial denaturation step of 5 min at 95℃, followed by 40 cycles of 25 s at 95℃, 55 s at 60℃ and 55 s at 72℃, and a final extension step of 7 min at 72℃. The 5′ ends of all reverse primers were labelled with biotin for chromogenic detection. Amplified PCR products were used in a REBA. Biotin labelled amplification products were automatically processed from denaturation for color development, and strip analysis began after installing the necessary reagents and equipment in the MolecuTech HybREAD480® instrument and pressing start. In the MolecuTech HybREAD480® system, the reaction proceeded as follows. First, PCR products were mixed with the denaturation solution. Denatured PCR products in hybridization solution (sodium chloride [NaCl], monosodium phosphate [NaH2PO4], ethylenediaminetetraacetic acid [EDTA], sodium lauroyl sarcosinate [SDS]) were incubated with REBA MTB-MDR membrane strips at 55℃. After washing with washing solution (NaCl, NaH_2_PO_4_, EDTA, SDS) at 55℃, the strips were incubated with streptavidin-conjugated alkaline phosphatase in conjugate dilution solution (Tris/hydrochloric acid [HCl]/NaCl/Tween 20) at room temperature, after which they were washed with conjugate dilution solution (Tris/HCl/NaCl/Tween 20). For the chromogenic reaction, the strips were incubated with nitro blue tetrazolium chloride and 5- bromo-4-chloro-3-indolyl phosphate in staining solution (Tris/HCl/NaCl/MgCl_2_/Tween 20). Following washing with distilled water at room temperature, the results were interpreted using the respective line assignment of the provided data sheet and the MolecuTech HybREAD480® (Supplementary Fig. S1).

### 2.5 Sequencing

Sanger sequencing was performed on DNA extracted from 174 *M.tuberculosis* isolates. Selected fragments of seven genetic loci containing hot spots for mutations associated with the resistance of tubercle bacilli to RIF (*rpoB*), INH (*katG*, *inhA* promoter) were PCR-amplified, and sequence variations were confirmed using ABI3730 in Bioneer (Daejeon, South Korea). Primers used to amplify gene targets for sequencing and amplification conditions are described in Supplementary Table S1.

### 2.6 Hain MTBDR*plus*

Mycobacterial DNA was extracted using a GenoLyse kit (Hain Lifescience, Nehren, Germany)-based manufacturer; we used 500 μL of decontaminated culture sample. PCR was performed using pre-made amplification mixes (amplification mix A and amplification mix B) that contained all the necessary components. Hybridization was performed using an automated GT Blot 48 device (Hain Lifescience, Nehren, Germany), and the results were interpreted based on the operating manual provided by the manufacturer.

### 2.7 Statistical analysis

The sensitivity, specificity, and diagnostic accuracy of the MolecuTech® REBA MTB-MDR kit were compared with the gold standard pDST. The precision of the estimates was reported using 95% confidence intervals (CIs). Concordance rates between the MolecuTech® REBA MTB-MDR kit and sequencing were calculated for the individual drug and drug groups.

## 3 Result

### 3.1 Evaluation of MolecuTech®REBA MTB-MDR performance

We evaluated the performance of the MolecuTech®REBA MTB-MDR (REBA) assay by comparing its results with those obtained from pDST using MGIT960 and DNA sequencing. The analysis included sensitivity, specificity, and concordance rates for the detection of resistance to INH and RIF (Table 1). For INH resistance detection, REBA demonstrated a sensitivity of 100% and a specificity of 100%. The assay showed a sensitivity of 100% and a specificity of 100% for RIF resistance detection. The overall concordance between REBA and pDST results was 100% (174/174), indicating a high level of agreement. Our results (100% for INH and 100% for RIF) are consistent with these findings, suggesting that REBA performs at the higher end of current molecular diagnostic capabilities.

**Table 1:** Diagnostic performance of MolecuTech®REBA MTB-MDR assays compared to phenotypic DST and gene sequencing.

	Anti-TB Drugs		pDST (MGIT)		Sensitivity (%) (95% CI)	Specificity (%) (95% CI)	Positive predictive value (PPV)(%)	Negative predictive value (NPV)(%)	Concordance pDST vs REBA (%)
			Susceptible	Resistant					
			No. of Isolates	No. of Isolates					
REBA	INH	Susceptible	96	0	100 (96.23–100.00)	100 (95.38–100)	100 (96.23–100)	100 (95.38–100)	100 (174/174)
		Resistant	0	78					
	RIF	Susceptible	96	0	100 (96.23–100.00)	100 (95.38–100)	100 (96.23–100)	100 (95.38–100)	100 (174/174)
		Resistant	0	78					

HAIN	INH	Susceptible	96	2	97.96 (92.82–99.75)	100 (95.26–100)	100 (96.23–100)	97.44 (90.60–99.34)	98.85 (172/174)
		Resistant	0	76					
	RIF	Susceptible	96	3	96.97 (91.40–99.37)	100 (95.20–100)	100 (96.23––100)	96.15 (89.13–98.70)	98.28 (171/174)
		Resistant	0	75					

SEQ	INH	Susceptible	96	0	100 (96.23–100.00)	100 (95.38–100)	100 (96.23–100)	100 (95.38–100)	100 (174/174)
		Resistant	0	78					
	RIF	Susceptible	96	0	100 (96.23–100.00)	100 (95.38–100)	100 (96.23–100)	100 (95.38–100)	100 (174/174)
		Resistant	0	78					

INH: Isoniazid.

RIF: Rifampicin.

SEQ: Sequencing.

CI: Confidence Interval.

REBA: Reverse Elution Blot Assay.

pDST: Phenotypic Drug Susceptibility Testing.

### 3.2 Evaluation of Hain MTBDR*plus* performance

We conducted a comparative analysis of the Hain MTBDR*plus* assay using the same set of strains and evaluation methods employed for the REBA assessment (Table 1). The performance of Hain was evaluated against pDST results. For INH resistance detection, Hain demonstrated a sensitivity of 97.96% and a specificity of 100%. The assay showed a sensitivity of 96.97% and a specificity of 100% for RIF resistance detection. The overall concordance between Hain and pDST results for each INH 98.85% (172/174) and RIF 98.28% (171/174) was indicating this level of agreement (Supplementary Table S2).

However, several discrepant results were observed. In the INH test, two strains (ID75 and ID173) yielded discrepant findings in the Hain assay resistance (Supplementary Table S3.A). In the RIF test, three strains (ID143, ID168, and ID171) also demonstrated discrepant results (Supplementary Table S3.B). For the INH-discordant strains, all were classified as susceptible by the Hain assay but as resistant by pDST (MGIT). Sequencing revealed that these strains harbored the 315 AGC-ACC mutation, which is known to confer INH resistance. Similarly, for the RIF-discordant strains, both were reported as susceptible by the Hain assay but resistant by pDST, and sequencing identified the 531 TCG-CAG mutation responsible for RIF resistance (Supplementary Table S3).

Overall, while the Hain MTBDR*plus* assay showed high sensitivity, specificity, and good concordance with pDST, these findings reveal some limitations of molecular testing. Specifically, certain common mutations causing INH and RIF resistance (such as 315 AGC-ACC and 531 TCG-CAG) may not be fully detected by the probes used, possibly due to probe design or interference from nearby DNA sequence variations affecting hybridization. This highlights the need for cautious interpretation of molecular test results and, where necessary, complementary phenotypic testing.

## 4 Discussion

The rapid and accurate detection of MDR-TB remains a critical challenge in global TB control efforts. Traditional solid culture methods, while reliable, are time-consuming and require specialized personnel, leading to significant delays in patient treatment. To address these limitations, molecular diagnostic methods have been developed to provide faster and more accurate drug susceptibility results. In this study, we evaluated the performance of the MolecuTech® REBA MTB-MDR kit, a molecular diagnostic tool developed by YD Diagnostics, for the detection of MDR-TB. Our findings demonstrate that the REBA MTB-MDR kit shows superior performance to the widely used Hain MTBDR*plus* assay, which is currently recommended by the WHO for rapid MDR-TB diagnosis. The MolecuTech® REBA MTB-MDR kit demonstrated high sensitivity and specificity for both INH and RIF resistance detection (Supplementary data Table S1). For INH, the sensitivity was 100% and specificity was 100%, while for RIF, the sensitivity was 100% and specificity was 100%. These results are consistent with or slightly better than those reported in recent *meta*-analyses of molecular diagnostic tools for MDR-TB detection [11], [12]. One notable improvement in the MolecuTech® REBA MTB-MDR kit is the inclusion of the *ahpC* gene target, which enhances the detection of INH resistance. This addition addresses a known limitation of some molecular assays, as mutations in *ahpC* can contribute to INH resistance in the absence of *katG* or *inhA* mutations [13], [14].

This study aimed to perform experiments using strains harboring mutations in the *ahpC* gene; however, relevant clinical isolates were not obtained due to the very low frequency of *ahpC* mutations in Korea. Molecular epidemiological studies of isoniazid (INH)-resistant *Mycobacterium tuberculosis* isolates in Korea have demonstrated that the major resistance mechanisms involve the *katG* 315 AGC-ACCmutation (approximately 70–80%) and *inhA* promoter mutations (approximately 15–25%), whereas *ahpC*-associated mutations are observed at a markedly lower frequency (<5%) [15], [16], [17]. This trend has been consistently reported across multiple domestic studies, suggesting that INH resistance due to *ahpC* mutations is rare in Korea. Therefore, additional experiments are required to verify the study results once *ahpC* mutant strains become available.

The MolecuTech® REBA MTB-MDR kit in combination with HybREAD480® offers an advantage in reducing human error and improving workflow efficiency. This aligns with the increasing trend towards automation in TB diagnostics to enhance reproducibility and reduce turnaround time [18], [19]. While our study focused on the laboratory performance of the kit using well-characterized strains, further research is needed to evaluate its performance with clinical samples. Recent studies have emphasized the importance of validating molecular assays in diverse clinical settings to ensure their real-world effectiveness [20], [21].

In conclusion, the MolecuTech® REBA MTB-MDR kit demonstrates promising performance for rapid MDR-TB detection, with potential to improve patient outcomes through earlier diagnosis and treatment initiation. However, as with all molecular tests, it should be used in conjunction with phenotypic testing and clinical judgment for optimal patient management.

## CRediT authorship contribution statement

**Eun-Soon Son:** Writing – review & editing, Writing – original draft, Validation, Formal analysis, Conceptualization. **Ji-im Lee:** Methodology, Formal analysis, Conceptualization. **Ji-Hoi Kim:** Validation, Software, Resources. **Eunjin Cho:** Project administration, Funding acquisition. **Mee-Kyung Kee:** Resources, Project administration. **SoonMog So:** Supervision, Software, Resources. **Jong Seok Lee:** Writing – review & editing, Supervision, Conceptualization.

## Declaration of competing interest

The authors declare that they have no known competing financial interests or personal relationships that could have appeared to influence the work reported in this paper.

## Appendix A Supplementary data

Supplementary Data 1 The REBA-MDR strip comprises 19 probes (13 wild-type and 6 mutant-type-specific probes) for the detection of RIF and INH resistance.

Supplementary Data 2 Overview of discordant cases and data among pDST, MolecuTech REBA MTB-MDR®, Hain MTBDRplus, and sequencing.
