# Supplementary material for: Evaluation of the laboratory performance of MolecuTech®REBA MTB-MDR kit for the detection of multidrug resistant tuberculosis
Source: J Clin Tuberc Other Mycobact Dis. 2026 May 6;44:100616. doi: 10.1016/j.jctube.2026.100616 (PMC13191270; doi:10.1016/j.jctube.2026.100616)
Supplement: Supplementary Data 1 — The REBA-MDR strip comprises 19 probes (13 wild-type and 6 mutant-type-specific probes) for the detection of RIF and INH resistance. [file mmc1.docx]

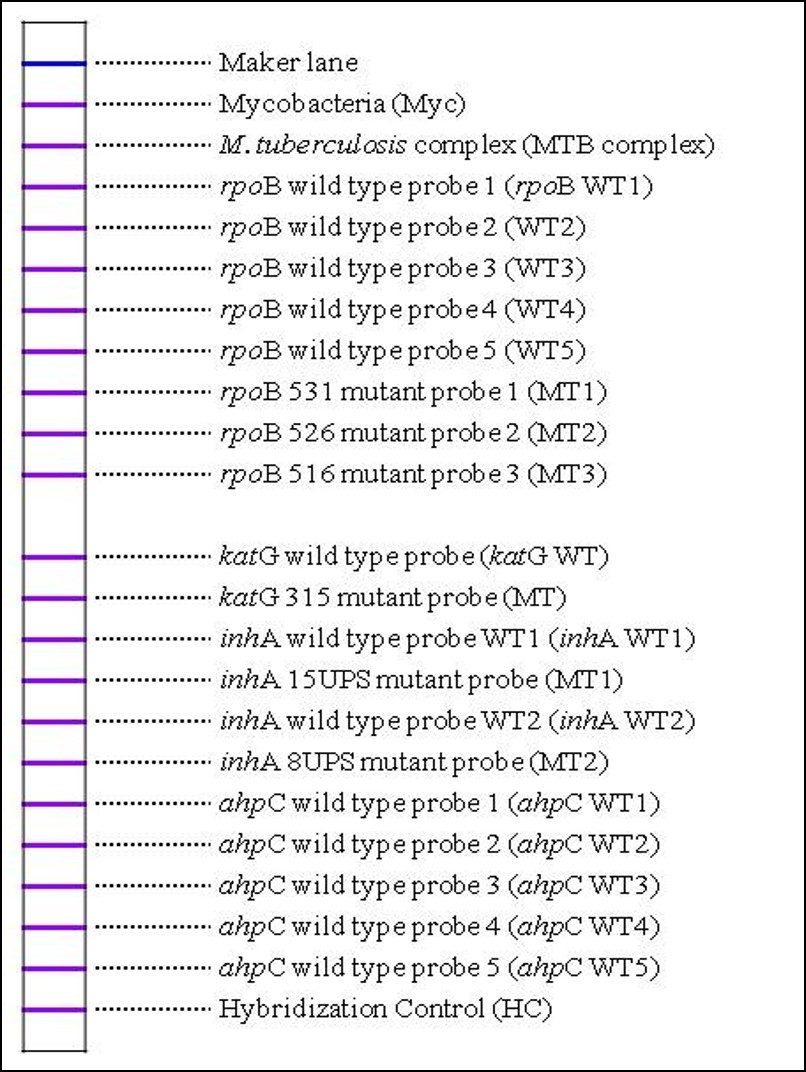


**Supplementary Figure 1. The REBA-MDR strip comprises 19 probes (13 wild-type and 6 mutant-type-specific probes) for the detection of RIF, INH (e.g., RIF and INH resistance).**
